# Prognostic factors for survival in patients with metastatic lung adenocarcinoma: An analysis of the SEER database

**DOI:** 10.1111/1759-7714.13681

**Published:** 2020-09-28

**Authors:** Begoña Campos‐Balea, Javier de Castro Carpeño, Bartomeu Massutí, David Vicente‐Baz, Diego Pérez Parente, Pedro Ruiz‐Gracia, Leonardo Crama, Manuel Cobo Dols

**Affiliations:** ^1^ Oncology, Hospital Universitario Lucus Augusti (HULA) Lugo Spain; ^2^ Oncology Hospital Universitario La Paz Madrid Spain; ^3^ Oncology Hospital Universitario Alicante (ISABIAL) Alicante Spain; ^4^ Oncology Hospital Universitario Virgen Macarena Seville Spain; ^5^ Lung Cancer. Medical Affairs Department, Roche Farma S.A Madrid Spain; ^6^ Medical Oncology, Unidad de Gestión Clínica Intercentros de Oncología Médica. Hospitales Universitarios Regional y Virgen de la Victoria. IBIMA Málaga Spain

**Keywords:** Lung adenocarcinoma, metastasis, non‐small cell lung cancer, overall survival, prognostic factor

## Abstract

**Background:**

Lung adenocarcinoma (ADC) is the main cause of death related to lung cancer. The aim of this study was to identify poor prognostic factors for overall survival (OS) in patients with stage IV lung ADC in real‐world clinical practice.

**Methods:**

Patients were selected from the Surveillance Epidemiology and End Results (SEER) database. Chi‐square bivariate analysis was used for the association of binary qualitative variables. A multivariate Cox regression analysis was performed to determine the impact of these prognostic factors on OS.

**Results:**

A total of 46 030 patients were included (51.3% men, mean age 67.03 ± 11.6), of whom 41.3% presented with metastases in bone, 28.9% in brain, 17.1% in liver and 31.8% in lung. Patients with liver metastases presented with two or more metastatic sites more frequently than patients without liver metastases (*P* < 0.001). Male sex (HR 0.78, 95% CI: 0.76–0.80), age ≥ 65 years (HR 1.37, 95% CI: 1.33–1.40), lack of family support (HR 0.80, 95% CI: 0.78–0.81) and presence of liver (HR 1.45, 95% CI: 1.40–1.50), bone (HR 1.21, 95% CI: 1.18–1.24) or brain metastases (HR 1.18, 95% CI: 1.15–1.21) were identified as poor prognostic factors for OS. Patients with liver metastasis showed the highest hazard ratio value (*P* < 0.001).

**Conclusions:**

The presence of liver metastases was the worst prognostic factor for patients with metastatic lung ADC. This factor should be considered as a stratification factor for future studies evaluating new cancer treatments including immunotherapy.

**Key points:**

**Significant findings of the study:**

- Regression analysis identified poor prognostic factors for overall survival. Factors were male sex, age ≥ 65 years, lack of family support and presence of liver, bone and brain metastases.
- Patients with liver metastasis showed the highest HR (HR = 1.45 95% CI: 1.40–1.50).
- This study included the highest number of adenocarcinoma patients analyzed so far (*N* = 46 030).

What this study adds

- The presence of liver metastases should be considered as a stratification factor for future studies evaluating new cancer treatments including immunotherapy.

## Graphical abstract

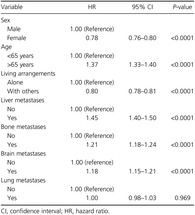

In this study we identified male sex, age = 65 years, lack of family support and liver and brain metastases as poor prognostic factors for overall survival (OS) in patients with stage IV lung ADC. The presence of liver metastases emerged as the worst prognostic factor. Consequently, it should be considered as a stratification factor for future studies evaluating new cancer treatments including immunotherapy.

## Introduction

Lung cancer (LC) is the leading cause of cancer‐related mortality worldwide. According to the WHO, of a total of 9.6 million cancer deaths in 2018, 1.76 million were due to LC.^1^ The main subtypes of LC based on histology are small cell lung carcinoma (SCLC) and non‐small cell lung carcinoma (NSCLC), accounting for 15% and 85% of all cases, respectively.^2^ NSCLC is further classified into three major histological subtypes: squamous cell carcinoma (SQCC), nonsquamous or adenocarcinoma (ADC) and large cell carcinoma.^3^ ADC is the most common subtype of NSCLC,^4^ and the most common type of LC in smokers and non‐smokers in men and women irrespective of age.^5^

Overall survival (OS) for all people with all types of LC at five years is 19%, with great variability and heterogeneity among the different subtypes, ranging from 24% for NSCLC patients to 6% in SCLC.^6^ Given this variability, the search for prognostic factors has been of great interest in the field. In NSCLC, prognostic factors include performance status measured on the Karnofsky scale or on the Eastern Cooperative Oncology Group (ECOG) scale, sex,^7^ tumor biology and molecular characteristics (histology grade, proliferation rate, pleural and vascular invasion, mutational status), treatment, age, smoking history, socioeconomic status, ethnicity, comorbidity, presence of pulmonary symptoms and weight loss.^8^ However, some of those have limited reproducibility (ie, histology), except in the case of neuroendocrine tumors which have the worst prognosis.^9^

Notably, the tumor‐node‐metastasis (TNM) classification system for the staging of cancer published by the Union for International Cancer Control (UICC)^10^ has been one of the most reproducible prognostic factors. Stage is then a powerful prognostic variable that integrates the information included in the three separate factors that per se are prognostic factors: tumor size (T) nodal (N) and metastatic (M) involvement (number of metastasis and location).^7^ Importantly, 70% of patients present with advanced‐stage disease at diagnosis.^11^ Nervous system, bone, liver, respiratory system and adrenal glands are the most common sites for LC metastasis, while bone metastasis is the most frequent in patients with ADC (39%).^12^ The prognosis and survival rate for patients with stage IVb disease is very poor, with a median overall survival (mOS) of five months in individuals with stage IV NSCLC. Specifically, patients with liver metastasis have the worst prognosis with an OS below three months.^13^

Prognostic factors can be used to construct homogenous groups of patients, and to obtain information about disease course, helping guide therapy in some cases by, for example, identifying subgroups of individuals in whom more aggressive therapies are required. They can also be used as stratification factors.^7^ However, an agreement on the set of factors that should systematically be used to adjust the effect of new factors is lacking. Data arising from clinical practice and not only from clinical trials are needed to confirm the power of certain variables as prognostic factors. With this perspective, the aim of this study was to identify poor prognostic factors for OS in patients with stage IV lung ADC in real‐world clinical practice.

## Methods

### Data collection

We used the Surveillance Epidemiology and End Results (SEER) database^14^ selecting all patients with stage IV lung ADC diagnosed between 2010 and 2015 with several metastatic sites (bone, brain, lung and liver), as well as those with multiple metastases. Patients with incomplete or missing information were excluded. Only distant metastatic lesions of the liver, brain, lung and bone were included. Other common sites, such as the pleura, adrenal gland and gastrointestinal tract were excluded. The inclusion codes and criteria from the SEER database were as follows: ADC (histological type: 8140), primary site (C34.1‐Lung) and derived AJCC stage group IV. Lung cancer staging determination was performed according to the seventh edition of the American Joint Committee on Cancer (AJCC) staging manual and the future of TNM.^15^

Demographic characteristics of patients (age, sex, living arrangements, marital status and race), as well as tumor location and mOS were collected. The presence of bone (not including the bone marrow), brain (not including the spinal cord or other parts of the central nervous system), lung (not including the pleura or pleural fluid) or liver metastasis was also reviewed. Survival time was considered as the time between diagnosis and death or the last follow‐up time according to SEER program definition. OS was the time from the date of diagnosis to death from any cause.

### Statistical analysis

Data are presented as frequencies (percent) or median deviation (range). Comparisons of continuous variables were performed using one‐way analysis of variance (ANOVA). Chi‐square bivariate analysis was used for the association of binary qualitative variables. The OS was analyzed using the Kaplan‐Meier method and the log‐rank test comparing survival in two or more groups. A multivariate Cox regression analysis was performed to determine the impact of these prognostic factors on OS. A two‐sided *P*‐value < 0.05 was considered statistically significant. All analyses were conducted using SEER Stat software v.8.3.6 (https://seer.cancer.gov/seerstat/).

## Results

### Patient characteristics

A total of 46 030 patients from the SEER database^14^ diagnosed between 2010 and 2015 were included in this study. Among all patients, 23 609 (51.3%) were men, 25 229 (54.8%) were ≥ 65 years old and 31 524 (68.6%) were white/non‐Hispanic. Most were married (*n* = 23 212, 52.9%) and lived with others (*n* = 23 316, 53.1%) (Table 1).

**Table 1:** Characteristics of study patients with stage IV lung adenocarcinoma

	*N* = 46 030
Age (years) mean (SD)	67.03 (11.6)
>65 years *n* (%)	25 229 (54.8)
Sex *n* (%)	
Men	23 609 (51.3)
Women	22 421 (48.7)
Race/ethnicity *n* (%)	
White non‐Hispanic	31 524 (68.6)
Black non‐Hispanic	5953 (13.0)
Asian/Pacific Islander	4814 (10.5)
Hispanic (all races)	3462 (7.5)
Indian/Alaska Native	213 (0.5)
Unknown	64 (0.1)
Marital status *n* (%)	
Single	7436 (16.9)
Married	23 212 (52.9)
Divorced/separated	6708 (13.9)
Widowed	7018 (16.0)
With partner	104 (0.2)
Unknown	2155 (4.7)
Living arrangements *n* (%)	
Alone	20 559 (46.9)
With others	23 316 (53.1)
Metastatic site *n* (%)	
Bone	18 329 (41.3)
Brain	12 811 (28.9)
Liver	7544 (17.1)

At diagnosis, 18329 (41.3%) patients presented with metastases in bone, 12 811 (28.9%) in brain, 7544 (17.1%) in liver and 13 935 (31.8%) in lung. In total, 19 722 (46.5%) patients had only one metastatic site, 8917 (21%) had two and 3700 (8.7%) had three or more metastatic sites (Table 1). Patients with liver metastases presented with two or more metastatic sites in brain, bone and lung more frequently than patients without liver metastases (2: 28.9% vs. 17.2%; 3: 9.4% vs. 2.9%; *P* < 0.001). Among patients with liver metastases, 78.5% had at least one other involved site (bone: 76.4%; lung: 47.1%; and brain: 37.2%). Furthermore, the mean number of metastatic sites was higher in patients with liver metastases (1.26 vs. 0.95; *P* < 0.001) (Table S1).

### Survival outcomes

**Figure 1:**
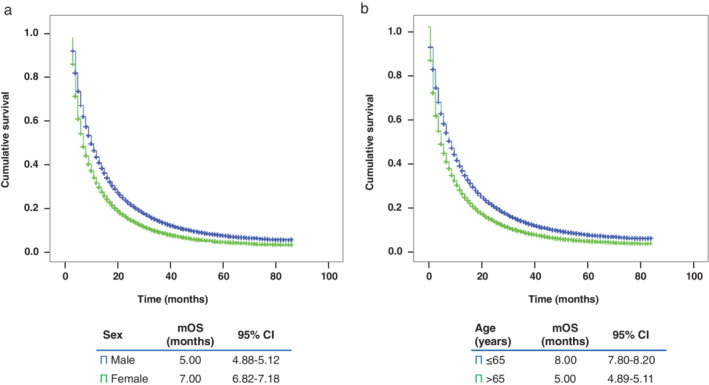
Kaplan‐Meier curve of overall survival based on (**a**) sex or (**b**) age. CI, confidence interval; mOS, median overall survival.

In the overall population, mOS was six (5.90–6.09) months. Specific survival analyses were performed to determine putative differences based on different patient characteristics. Thus, worse mOS was found among men (5 months, 95% CI: 4.88–5.12 vs. 7 months 95% CI: 6.82–7.18, *P* < 0.001) (Fig 1a and Table S2) and in patients ≥65 years (5 months, 95% CI: 4.89–5.11 vs. 8 months 95% CI: 7.80–8.20, *P* < 0.001) (Fig 1b). Among race/ethnicity characteristics, patients identified as Asian or Pacific Islander showed the highest mOS (11 months 95% CI: 10.37–11.63, *P* < 0.001) (Figure S1). In contrast, patients lacking family support had worse mOS than patients living with others (5 months 95% CI: 4.88–5.11 vs. 7 months 95% CI: 6.83–7.17, *p* < 0.001) (Figure S2).

**Figure 2:**
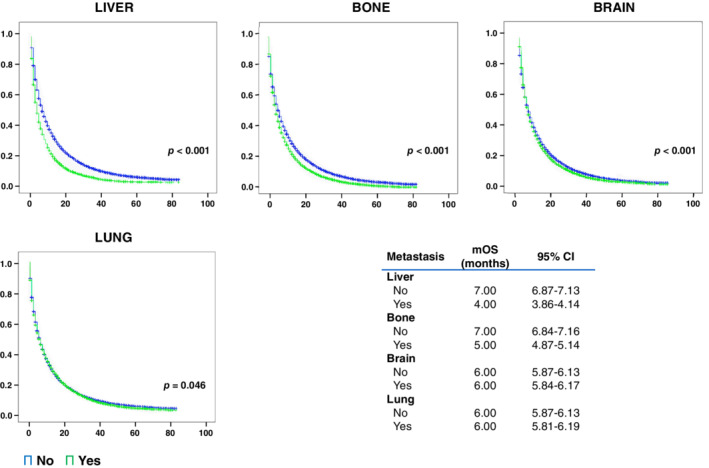
Survival of patients by metastatic site. CI, confidence interval; mOS, median overall survival (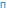) No, and (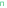) Yes.

**Figure 3:**
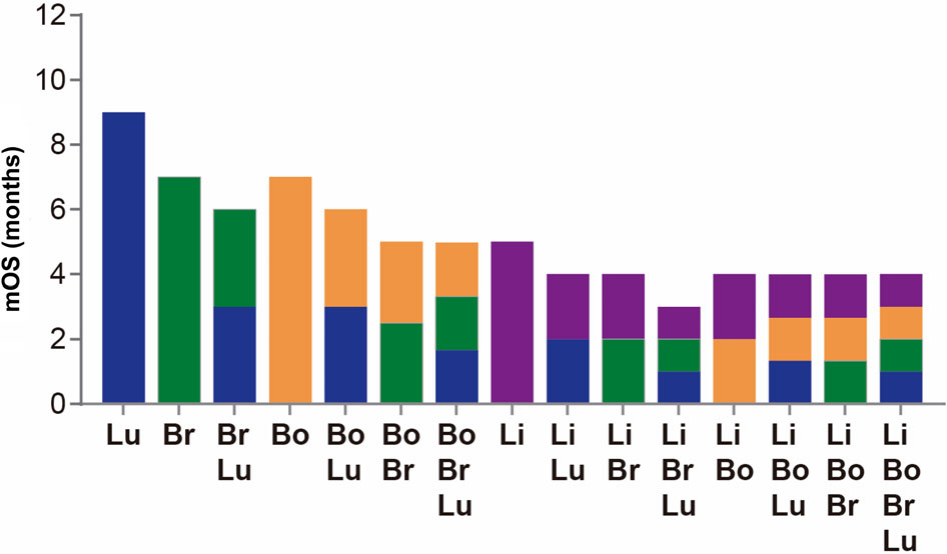
Survival of patients by metastatic site combination. mOS, median overall survival (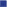) Lung (Lu), (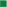) Brian (Br), (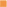) Bone (Bo), and (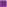) Liver (Li).

OS was also assessed based on the site of metastases. The median OS for patients with NSCLC with liver, bone, brain and lung metastases was four, five, and six months, respectively (Fig 2). Additionally, patients with liver metastases had worse one‐, two‐, and five‐year survival probability than those without liver metastases (Figure S3). For patients with only one metastatic site, those with liver metastases showed the lowest mOS values (5 months, 95% CI: 4.48–5.53), followed by bone (7 months, 95% CI: 6.73–7.27) and brain (7 months, 95% CI: 6.70–7.30). The highest values were found in patients with only one metastatic site in the lung (9 months, 95% CI: 8.55–9.45) (Fig 3). Patients with two or more metastatic sites showed the worst mOS (≤4 months) only if liver metastases were present (Table 2), while the presence of liver, brain and lung metastasis comprised the combination of metastatic sites with the worst mOS value (3 months, 95% CI: 2.39–3.62) (Fig 3).

**Table 2:** Survival of patients by metastatic site combination

Metastatic site combination	*n* (%)	mOS	95% CI
Unknown	10 059 (23.7)	7.0	6.72–7.28
Lung	5538 (13.1)	9.0	8.55–9.44
Brain	5463 (12.9)	7.0	6.70–7.30
Lung + Brain	1433 (3.4)	6.0	5.50–6.50
Bone	7218 (17.0)	7.0	6.73–7.27
Lung + Bone	2536 (6.0)	6.0	5.57–6.43
Brain + Bone	2136 (5.0)	5.0	4.65–5.36
Lung + Brain + Bone	1027 (2.4)	5.0	4.51–5.49
Liver	1503 (3.5)	5.0	4.47–5.53
Lung + Liver	645 (1.5)	4.0	3.41–4.59
Brain + Liver	424 (1.0)	4.0	3.54–4.46
Lung + Brain + Liver	227 (0.5)	3.0	2.39–3.61
Bone + Liver	1743 (4.1)	4.0	3.72–4.23
Lung + Bone+ Liver	1055 (2.5)	4.0	3.67–4.33
Brain + Bone + Liver	735 (1.7)	4.0	3.50–4.50
Lung + Brain + Bone + Liver	656 (1.5)	4.0	3.51–4.49

CI, confidence interval; mOS, median overall survival.

Multivariate Cox proportional hazard models were used to identify prognostic factors in patients with lung ADC (Table 3 **).** The analysis revealed that male sex, age ≥ 65, lack of family support and presence of liver, bone or brain metastases were poor prognostic factors for OS. Overall, OS was mostly affected by the presence of liver metastases (HR = 1.45 95% CI: 1.40–1.50), age ≥ 65 years (HR = 1.37 95% CI: 1.33–1.40), and bone metastases (HR = 1.21 95% CI: 1.18–1.24).

**Table 3:** Multivariate Cox regression analysis for overall survival (OS) in patients with metastatic lung adenocarcinoma

Variable	HR	95% CI	*P*‐value
Sex			
Male	1.00 (Reference)		
Female	0.78	0.76–0.80	<0.0001
Age			
<65 years	1.00 (Reference)		
>65 years	1.37	1.33–1.40	<0.0001
Living arrangements			
Alone	1.00 (Reference)		
With others	0.80	0.78–0.81	<0.0001
Liver metastases			
No	1.00 (Reference)		
Yes	1.45	1.40–1.50	<0.0001
Bone metastases			
No	1.00 (Reference)		
Yes	1.21	1.18–1.24	<0.0001
Brain metastases			
No	1.00 (reference)		
Yes	1.18	1.15–1.21	<0.0001
Lung metastases			
No	1.00 (Reference)		
Yes	1.00	0.98–1.03	0.969

CI, confidence interval; HR, hazard ratio.

## Discussion

NSCLC is frequently diagnosed at advanced stages. Five‐year survival rates in advanced disease are typically poor when chemotherapeutic‐based strategies are implemented,^16^ but they have increased in recent years thanks to new therapeutic approaches such as targeted therapies and immunotherapy.^17^ Prognostic factors must be identified to design individualized treatment plans that not only improve efficacy outcomes and patients’ quality of life, but also reduce the incidence of adverse effects.^18^ In this setting, then, we identified the main factors affecting the OS of stage IV lung ADC patients in the largest database to date (SEER database^14^).

Our results first reveal clear differences associated with patient baseline characteristics such as sex, age, ethnicity and living arrangements. Women had longer mOS than men, and this observation has been reported in several studies in which NSCLC female patients demonstrated a decreased risk of progression and death even after adjusting for age, histology and stage.^19^, ^20^, ^21^, ^22^ Indeed, the comparison of several studies in a meta‐analysis confirmed that some targeted treatments were influenced by sex,^23^ and one hypothesis is that the better survival outcomes shown by women may be due to different hormone and receptor expression levels.^24^ Regarding age, our results that show better mOS values for patients younger than 65 years of age are in line with previous studies, in which age has been clearly identified as a factor affecting survival in NSCLC patients.^25^, ^26^, ^27^ Other characteristics, such as living arrangements, also confirm previous data suggesting that living with others, along with the corresponding help and support, are associated with longer mOS compared to individuals who lack this support.^25^, ^28^, ^29^ Finally, our study reveals differences in terms of ethnicity, with the lowest value found in Asian/Pacific Islanders with NSCLC. Although other authors have reported similar results with better outcomes in Asian patients compared to Caucasian patients,^25^, ^30^ this topic remains controversial.^30^, ^31^, ^32^, ^33^ The higher proportion of oncogenic driver mutations found in Asian patients,^34^, ^35^, ^36^ subsequently reflected in better efficacy outcomes with EGFR tyrosine kinase inhibitors (TKIs),^37^, ^38^, ^39^, ^40^, ^41^ could explain these discrepancies. Finally, multivariate Cox regression analysis confirmed that female sex, age under 65 years and living with others were indeed prognostic factors for OS.

In NSCLC patients, the most frequent metastatic sites are brain, bone, liver, respiratory system, and adrenal glands.^42^, ^43^ In our study, the lowest mOS were reported in patients with liver metastases, the results of which were in accordance with previous studies.^12^, ^42^, ^43^, ^44^, ^45^, ^46^ Nakazawa *et al*. reported a 2.41‐fold higher mortality risk with liver metastasis compared to other distant metastases (*P* < 0.001).^42^ Riihimaki *et al*. found that the mortality risk with liver metastasis was 1.53‐fold higher than with brain metastasis (*P* < 0.05),^12^ while Tamura *et al*. registered a 1.55‐fold higher mortality risk with liver metastasis in comparison with other distant metastases (*P* < 0.001).^43^ We also observed poorer mOS values in patients with two or more metastatic sites, in line with previous data suggesting that the presence of multiple metastatic sites considerably reduced survival expectations.^45^, ^46^ Specifically, in our study these low values were only observed in patients whose metastatic site combinations involved the liver. In fact, patients with liver metastasis usually present a high disease burden.^12^, ^47^ Furthermore, the presence of liver, brain and lung metastasis was identified as the metastatic site combination with the worst mOS value (3 months, 95% CI: 2.39–3.62). However, the sequence of metastasis appearance and its impact on patient outcomes and survival could not be assessed, since the SEER database only provided information on patients' status at diagnosis. Despite this, our results clearly highlight the negative effect exerted by the presence of metastases in this organ and its relevance as a poor prognostic factor in NSCLC patients, especially in patients with ADC as observed in other studies.^13^ Indeed, our data confirmed a worse one‐, two‐ and five‐year survival probability in patients with liver metastases compared to other patients. Further analysis by multivariate Cox regression models confirmed that the presence of liver, brain and bone metastasis, but not lung metastasis, were poor prognostic factors for OS, to the extent that liver metastases emerged as the factor with the worst prognostic value (higher HR). At present, chemotherapy in combination with immunotherapy is the standard treatment for liver metastasis.^48^ In contrast to bone or brain involvement, few studies have focused on liver metastasis of lung cancer. Given its clear negative impact on survival, this form of distant disease should be specially considered as a stratification factor in future therapeutic clinical trials.

This study has certain limitations. First, the retrospective nature of the study limits its conclusions, and it was impossible to confidently exclude confounding factors, such as smoking history, mutational status, PD‐L1 expression levels, lactate dehydrogenase (LDH) and treatments received. Second, some significant patient data were omitted in the SEER program, such as specific chemotherapy regimens and tumor mutational status, all of which may have had an impact on patient prognosis. This information will need to be collected in future prospective studies.

In conclusion, in stage IV lung ADC patients from the SEER database, increased age, male sex and lack of family support without support were significantly associated with poor mOS, while the presence of metastases, and specifically the presence of liver metastases, were identified as risk factors for death.

## Disclosure

B.C‐B reports advisory and consultancy honoraria from Boehringer and Sanofi, speaker honoraria from Roche, Merck, Bristol‐Myers Squibb and AstraZeneca, as well as travel/accommodation/expenses support from Roche, Lilly and Boehringer.

J.C‐C. reports advisory and consultancy honoraria from Roche, Merck, Bristol‐Myers Squibb, AstraZeneca, Pfizer, PharmaMar, Boehringer, Takeda and Tesaro, speaker honoraria from Roche, Merck and AstraZeneca and travel/accommodation/expenses support from Roche, Merck, Bristol‐Myers Squibb and AstraZeneca.

B.M reports advisory and consultancy honoraria from Roche, Merck, AstraZeneca, Boehringer and Takeda, as well as speaker honoraria from Roche, Merck, Bristol‐Myers Squibb, Pfizer and Boehringer.

V.B reports advisory and consultancy honoraria from Roche, Merck, Bristol‐Myers Squibb, AstraZeneca, Pfizer, Boehringer and Takeda, as well as speaker honoraria from Roche, Merck, Bristol‐Myers Squibb, AstraZeneca, Pfizer and Boehringer.

M.C.D. reports advisory and consultancy honoraria from Roche, Bristol‐Myers Squibb, AstraZeneca, Pfizer and Boehringer and travel/accommodation/expenses support from Roche, Bristol‐Myers Squibb and AstraZeneca.

D.P.P, L.C. and P. R‐G. were full‐time employees of Roche Farma S.A. at the time the study was conducted.

## Supporting information


**Figure S1** Kaplan‐Meier curve of overall survival based on ethnicity. CI, confidence interval; mOS, median overall survival.
**Figure S2.** Kaplan‐Meier curve of overall survival based on living arrangements. CI, confidence interval; mOS, median overall survival.
**Figure S3.** Overall survival probability based on the presence of liver metastases.

**Table S1** Comparisons of patients with or without liver metastases.
**Table S2.** Presence of metastases according to sex.

## References

[1] World Health Organization (WHO) . Cancer. [Cited April 30, 2020.] Available from URL: https://webcache.googleusercontent.com/search?q=cache:R9_054PCc_EJ:https://www.who.int/news-room/fact-sheets/detail/cancer+&cd=1&hl=es&ct=clnk&gl=es.

[2] Sher T , Dy GK , Adjei AA . Small cell lung cancer. Mayo Clin Proc 2008; 83 (3): 355–67. 18316005 10.4065/83.3.355

[3] Travis WD , Brambilla E , Nicholson AG *et al* The 2015 World Health Organization classification of lung tumors: Impact of genetic, clinical and radiologic advances since the 2004 classification. J Thorac Oncol 2015; 10 (9): 1243–60. 26291008 10.1097/JTO.0000000000000630

[4] Imielinski M , Berger AH , Hammerman PS *et al* Mapping the hallmarks of lung adenocarcinoma with massively parallel sequencing. Cell 2012; 150 (6): 1107–20. 22980975 10.1016/j.cell.2012.08.029 PMC3557932

[5] Couraud S , Zalcman G , Milleron B , Morin F , Souquet PJ . Lung cancer in never smokers: A review. Eur J Cancer 2012; 48 (9): 1299–311. 22464348 10.1016/j.ejca.2012.03.007

[6] American Cancer Society . Cancer facts & figures. [Cited Apr 30 2020.] Available from URL: https://www.cancer.org/content/dam/cancer-org/research/cancer-facts-and-statistics/annual-cancer-facts-and-figures/2020/cancer-facts-and-figures-2020.pdf.

[7] Paesmans M . Prognostic and predictive factors for lung cancer. Breathe 2012; 9 (2): 112–21.

[8] Radkiewicz C , Dickman PW , Johansson ALV , Wagenius G , Edgren G , Lambe M . Sex and survival in non‐small cell lung cancer: A nationwide cohort study. PLOS One 2019; 14 (6): e0219206. 31247015 10.1371/journal.pone.0219206 PMC6597110

[9] Sanchez de Cos Escuin J . Diagnosis and treatment of neuroendocrine lung tumors. Arch Bronconeumol 2014; 50 (9): 392–6. 24685201 10.1016/j.arbres.2014.02.004

[10] Brierley JGM , Wittekind CH . TNM Classification of Malignant Tumours, 8th edn Wiley‐Blackwell, Oxford 2007.

[11] Travis WD , Brambilla E , Riely GJ . New pathologic classification of lung cancer: Relevance for clinical practice and clinical trials. J Clin Oncol 2013; 31 (8): 992–1001. 23401443 10.1200/JCO.2012.46.9270

[12] Riihimaki M , Hemminki A , Fallah M *et al* Metastatic sites and survival in lung cancer. Lung Cancer 2014; 86 (1): 78–84. 25130083 10.1016/j.lungcan.2014.07.020

[13] Ren Y , Dai C , Zheng H *et al* Prognostic effect of liver metastasis in lung cancer patients with distant metastasis. Oncotarget 2016; 7 (33): 53245–53. 27449299 10.18632/oncotarget.10644 PMC5288182

[14] National Cancer Institute . Surveillance, epidemiology, and end results (SEER). [Cited Apr 30 2020.] Available from URL: https://seer.cancer.gov/.

[15] Edge SB , Compton CC . The American Joint Committee on Cancer: The 7th edition of the AJCC cancer staging manual and the future of TNM. Ann Surg Oncol 2010; 17 (6): 1471–4. 20180029 10.1245/s10434-010-0985-4

[16] Dafni U , Tsourti Z , Vervita K , Peters S . Immune checkpoint inhibitors, alone or in combination with chemotherapy, as first‐line treatment for advanced non‐small cell lung cancer. A systematic review and network meta‐analysis. Lung Cancer 2019; 134: 127–40. 31319971 10.1016/j.lungcan.2019.05.029

[17] Goldstraw P , Chansky K , Crowley J *et al* The IASLC lung cancer staging project: Proposals for revision of the TNM stage groupings in the forthcoming (eighth) edition of the TNM classification for lung cancer. J Thorac Oncol 2016; 11 (1): 39–51. 26762738 10.1016/j.jtho.2015.09.009

[18] Reck M , Rabe KF . Precision diagnosis and treatment for advanced non‐small‐cell lung cancer. N Engl J Med 2017; 377 (9): 849–61. 28854088 10.1056/NEJMra1703413

[19] de Perrot M , Licker M , Bouchardy C , Usel M , Robert J , Spiliopoulos A . Sex differences in presentation, management, and prognosis of patients with non‐small cell lung carcinoma. J Thorac Cardiovasc Surg 2000; 119 (1): 21–6. 10612756 10.1016/s0022-5223(00)70213-3

[20] Hsu LH , Chu NM , Liu CC *et al* Sex‐associated differences in non‐small cell lung cancer in the new era: Is gender an independent prognostic factor? Lung Cancer 2009; 66 (2): 262–7. 19299032 10.1016/j.lungcan.2009.01.020

[21] Radzikowska E , Glaz P , Roszkowski K . Lung cancer in women: Age, smoking, histology, performance status, stage, initial treatment and survival. Population‐based study of 20,561 cases. Ann Oncol 2002; 13 (7): 1087–93. 12176788 10.1093/annonc/mdf187

[22] Visbal AL , Williams BA , Nichols FC III *et al* Gender differences in non‐small‐cell lung cancer survival: An analysis of 4,618 patients diagnosed between 1997 and 2002. Ann Thorac Surg 2004; 78 (1): 209–15 discussion 15. 15223430 10.1016/j.athoracsur.2003.11.021

[23] Pinto JA , Vallejos CS , Raez LE *et al* Gender and outcomes in non‐small cell lung cancer: An old prognostic variable comes back for targeted therapy and immunotherapy? ESMO Open 2018; 3 (3): e000344–e. 29682332 10.1136/esmoopen-2018-000344 PMC5905840

[24] Cheng TD , Darke AK , Redman MW *et al* Smoking, sex, and non‐small cell lung cancer: Steroid hormone receptors in tumor tissue (s0424). J Natl Cancer Inst 2018; 110 (7): 734–42. 29346580 10.1093/jnci/djx260 PMC6037104

[25] Liao Y , Fan X , Wang X . Effects of different metastasis patterns, surgery and other factors on the prognosis of patients with stage iv non‐small cell lung cancer: A surveillance, epidemiology, and end results (SEER) linked database analysis. Oncol Lett 2019; 18 (1): 581–92. 31289530 10.3892/ol.2019.10373 PMC6546983

[26] Tas F , Ciftci R , Kilic L *et al* Age is a prognostic factor affecting survival in lung cancer patients. Oncol Lett 2013; 6 (5): 1507–13. 24179550 10.3892/ol.2013.1566 PMC3813578

[27] Toffart AC , Duruisseaux M , Brichon PY *et al* Operation and chemotherapy: Prognostic factors for lung cancer with one synchronous metastasis. Ann Thorac Surg 2018; 105 (3): 957–65. 29397931 10.1016/j.athoracsur.2017.10.040

[28] Merrill RM , Johnson E . Benefits of marriage on relative and conditional relative cancer survival differ between males and females in the USA. J Cancer Surviv 2017; 11 (5): 578–89. 28770444 10.1007/s11764-017-0627-y

[29] Wu Y , Ai Z , Xu G . Marital status and survival in patients with non‐small cell lung cancer: An analysis of 70,006 patients in the SEER database. Oncotarget 2017; 8 (61): 103518–34. 29262581 10.18632/oncotarget.21568 PMC5732747

[30] Tannenbaum SL , Koru‐Sengul T , Zhao W , Miao F , Byrne MM . Survival disparities in non‐small cell lung cancer by race, ethnicity, and socioeconomic status. Cancer J 2014; 20 (4): 237–45. 25098282 10.1097/PPO.0000000000000058

[31] Richards TB , Henley SJ , Puckett MC *et al* Lung cancer survival in the United States by race and stage (2001‐2009): Findings from the concord‐2 study. Cancer 2017; 123 (Suppl 24)): 5079–99. 29205305 10.1002/cncr.31029 PMC5812722

[32] Videtic GM , Reddy CA , Chao ST *et al* Gender, race, and survival: A study in non‐small‐cell lung cancer brain metastases patients utilizing the radiation therapy oncology group recursive partitioning analysis classification. Int J Radiat Oncol Biol Phys 2009; 75 (4): 1141–7. 19327899 10.1016/j.ijrobp.2008.12.022

[33] Williams CD , Salama JK , Moghanaki D , Karas TZ , Kelley MJ . Impact of race on treatment and survival among U.S. Veterans with early‐stage lung cancer. J Thorac Oncol 2016; 11 (10): 1672–81. 27296104 10.1016/j.jtho.2016.05.030

[34] Steuer CE , Behera M , Berry L *et al* Role of race in oncogenic driver prevalence and outcomes in lung adenocarcinoma: Results from the lung cancer mutation consortium. Cancer 2016; 122 (5): 766–72. 26695526 10.1002/cncr.29812 PMC5038591

[35] Dearden S , Stevens J , Wu YL , Blowers D . Mutation incidence and coincidence in non small‐cell lung cancer: Meta‐analyses by ethnicity and histology (mutmap). Ann Oncol 2013; 24 (9): 2371–6. 23723294 10.1093/annonc/mdt205 PMC3755331

[36] Peng L , Wu YL . Immunotherapy in the Asiatic population: Any differences from Caucasian population? J Thorac Dis 2018; 10 (Suppl. 13): S1482–s93. 29951300 10.21037/jtd.2018.05.106 PMC5994501

[37] Cho BC , Chewaskulyong B , Lee KH *et al* Osimertinib versus standard of care EGFR TKI as first‐line treatment in patients with EGFRm advanced NSCLC: Flaura asian subset. J Thorac Oncol 2019; 14 (1): 99–106. 30240852 10.1016/j.jtho.2018.09.004

[38] Park K , Goto K . A review of the benefit‐risk profile of gefitinib in asian patients with advanced non‐small‐cell lung cancer. Curr Med Res Opin 2006; 22 (3): 561–73. 16574039 10.1185/030079906X89847

[39] Soria JC , Ohe Y , Vansteenkiste J *et al* Osimertinib in untreated EGFR‐mutated advanced non‐small‐cell lung cancer. N Engl J Med 2018; 378 (2): 113–25. 29151359 10.1056/NEJMoa1713137

[40] Wu YL , Saijo N , Thongprasert S *et al* Efficacy according to blind independent central review: Post‐hoc analyses from the phase iii, randomized, multicenter, IPASS study of first‐line gefitinib versus carboplatin/paclitaxel in Asian patients with EGFR mutation‐positive advanced NSCLC. Lung Cancer 2017; 104: 119–25. 28212993 10.1016/j.lungcan.2016.11.022

[41] Wu YL , Zhong WZ , Li LY a . Epidermal growth factor receptor mutations and their correlation with gefitinib therapy in patients with non‐small cell lung cancer: A meta‐analysis based on updated individual patient data from six medical centers in mainland China. J Thorac Oncol 2007; 2 (5): 430–9. 17473659 10.1097/01.JTO.0000268677.87496.4c

[42] Nakazawa K , Kurishima K , Tamura T *et al* Specific organ metastases and survival in small cell lung cancer. Oncol Lett 2012; 4 (4): 617–20. 23205072 10.3892/ol.2012.792 PMC3506697

[43] Tamura T , Kurishima K , Nakazawa K *et al* Specific organ metastases and survival in metastatic non‐small‐cell lung cancer. Mol Clin Oncol 2015; 3 (1): 217–21. 25469298 10.3892/mco.2014.410 PMC4251107

[44] Hoering A , Leblanc M , Crowley JJ . Randomized phase iii clinical trial designs for targeted agents. Clin Cancer Res 2008; 14 (14): 4358–67. 18628448 10.1158/1078-0432.CCR-08-0288 PMC2569946

[45] Gibson AJW , Li H , D'Silva A *et al* Impact of number versus location of metastases on survival in stage iv m1b non‐small cell lung cancer. Med Oncol 2018; 35 (9): 117. 30073425 10.1007/s12032-018-1182-8

[46] Yang J , Zhang Y , Sun X *et al* The prognostic value of multiorgan metastases in patients with non‐small cell lung cancer and its variants: A seer‐based study. J Cancer Res Clin Oncol 2018; 144 (9): 1835–42. 30003315 10.1007/s00432-018-2702-9 PMC11813380

[47] Shiroyama T , Suzuki H , Tamiya M *et al* Clinical characteristics of liver metastasis in nivolumab‐treated patients with non‐small cell lung cancer. Anticancer Res 2018; 3 (8): 4723–9. 10.21873/anticanres.12779 30061241

[48] Planchard D , Popat S , Kerr K *et al* Metastatic non‐small‐cell lung cancer: Esmo clinical practice guidelines for diagnosis, treatment and follow‐up (updated version september 2019). [Cited May 2020]. Available from: https://www.esmo.org/content/download/227453/3874538/1.

