# Supplementary material for: Prognostic factors for survival in patients with metastatic lung adenocarcinoma: An analysis of the SEER database
Source: Thorac Cancer. 2020 Sep 28;11(11):3357–64. doi: 10.1111/1759-7714.13681 (PMC7606019; doi:10.1111/1759-7714.13681)
Supplement: Supplementary file 1 — Figure S1 Kaplan‐Meier curve of overall survival based on ethnicity. CI, confidence interval; mOS, median overall survival. Figure S2. Kaplan‐Meier curve of overall survival based on living arrangements. CI, confidence interval; mOS, median overall survival. Figure S3. Overall survival probability based on the presence of liver metastases. [file TCA-11-3357-s001.pdf]

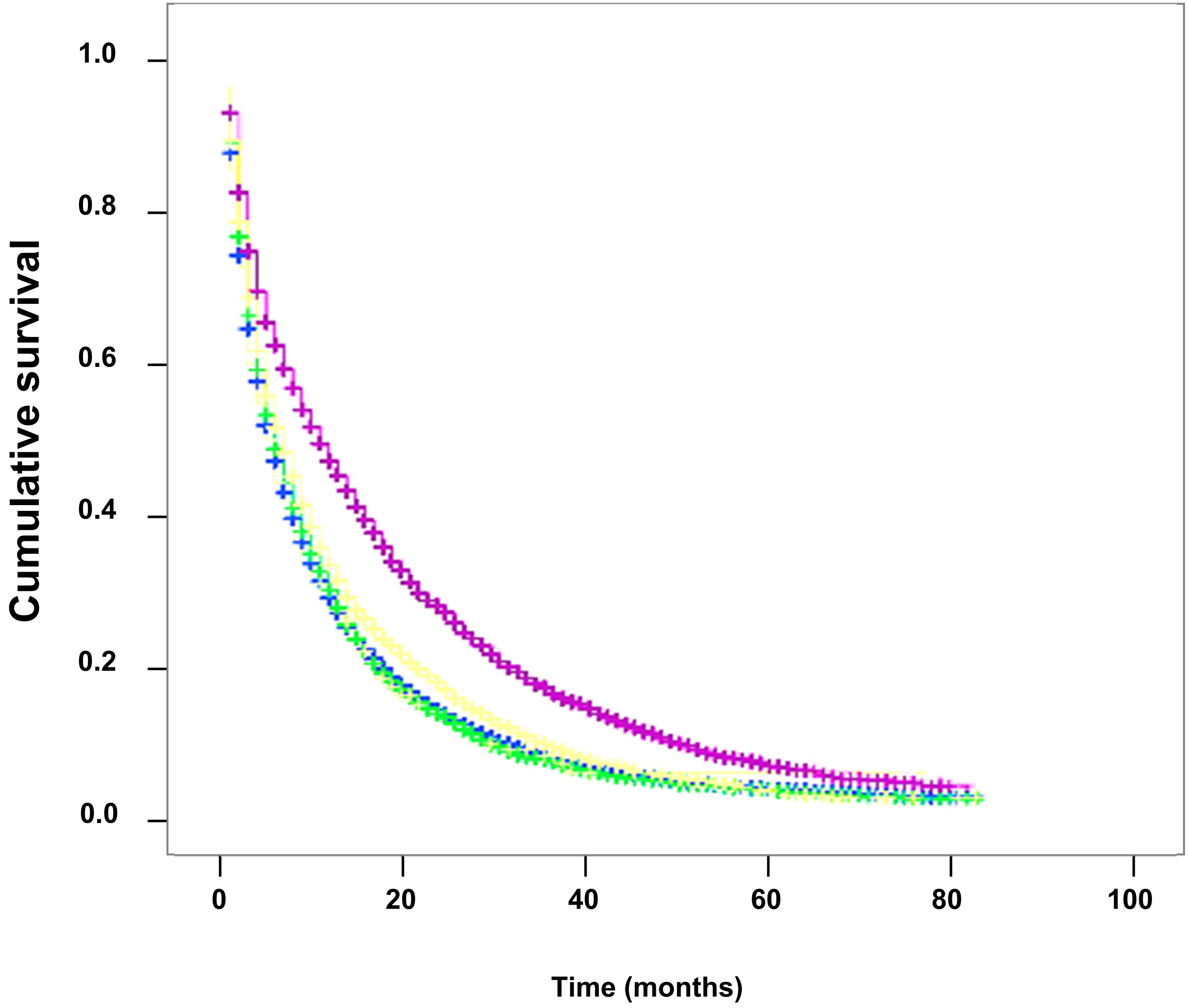

Race/ethnicity	mOS (months)	95% CI
White. non-Hispanic	6.00	5.89-6.11
Black. non-Hispanic	6.00	5.74-6.26
Asian/Pacific Islander	11.00	10.37-11.63
Hispanic	7.00	6.52-7.48
Indian/Alaska Native	5.00	3.70-6.30

Supplementary Fig. S1

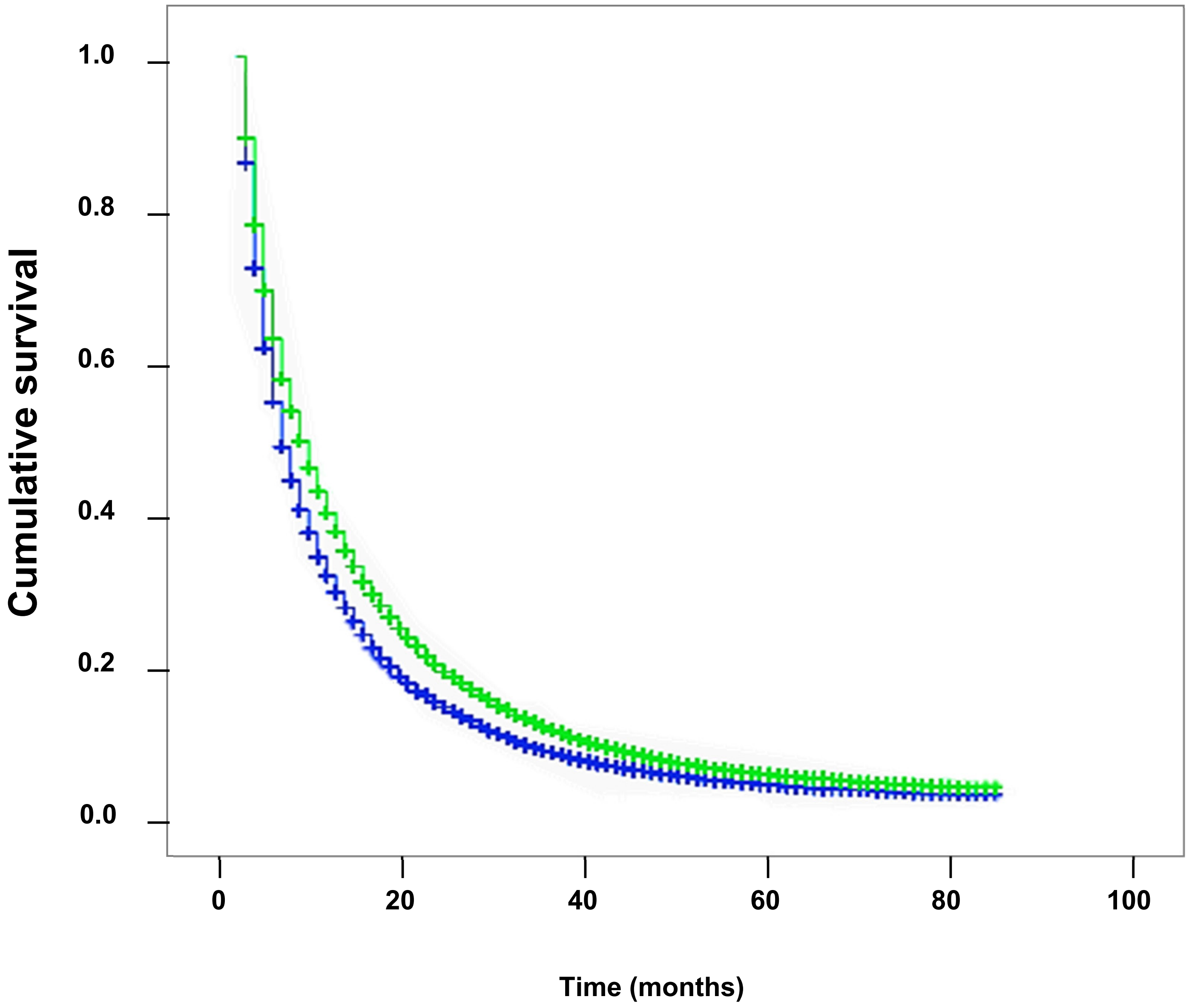

Living arrangements	mOS (months)	95% CI
Alone	5.00	4.88-5.12
With others	7.00	6.83-7.17

Supplementary Fig. S2

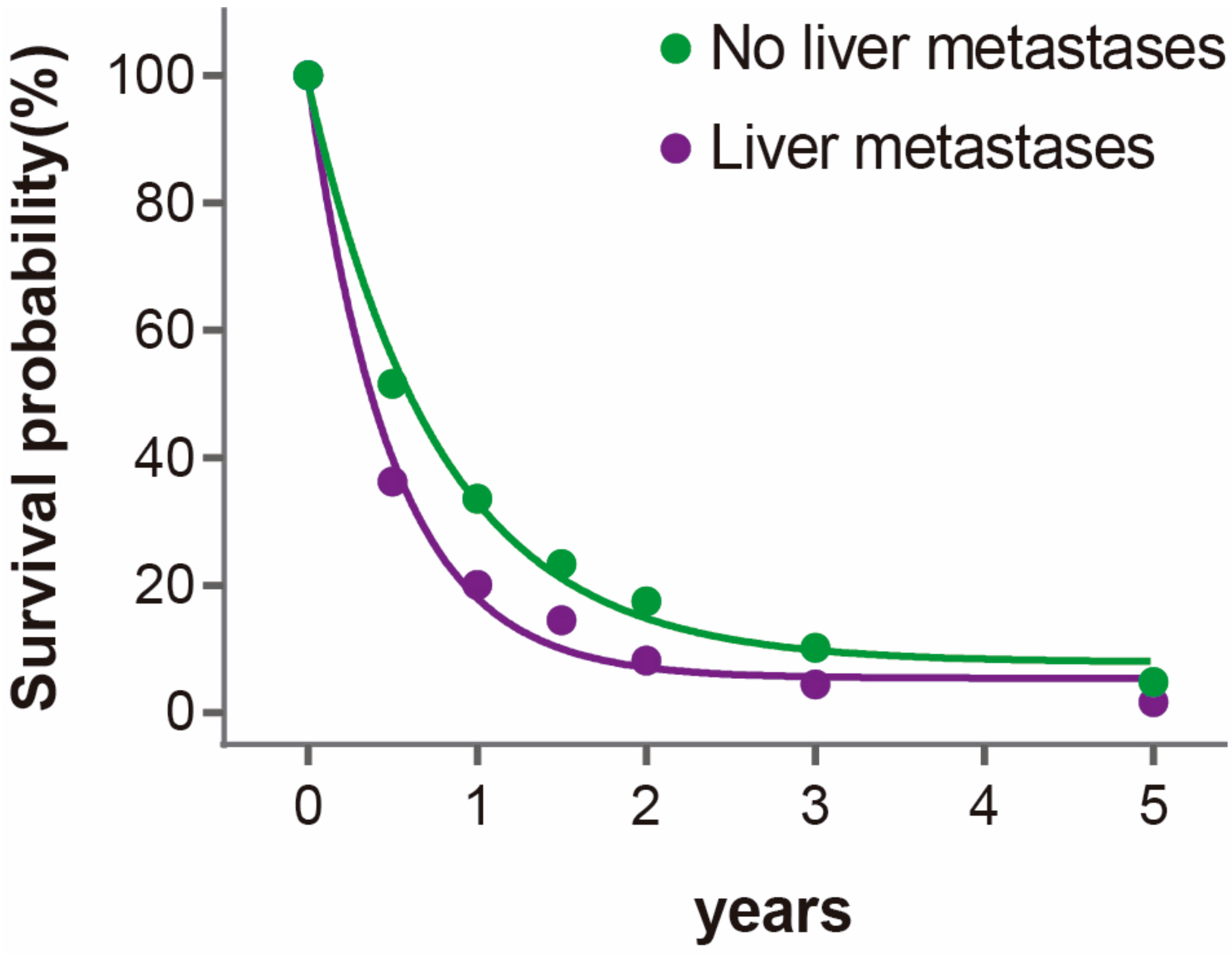

Supplementary Fig. S3
