# Supplementary material for: Prognostic factors for survival in patients with metastatic lung adenocarcinoma: An analysis of the SEER database
Source: Thorac Cancer. 2020 Sep 28;11(11):3357–64. doi: 10.1111/1759-7714.13681 (PMC7606019; doi:10.1111/1759-7714.13681)
Supplement: Supplementary file 2 — Table S1 Comparisons of patients with or without liver metastases. Table S2. Presence of metastases according to sex. [file TCA-11-3357-s002.docx]

**Supplementary Table S1.** Comparisons of patients with or without liver metastases

| **Clinical factor** | **No liver metastases** | **Liver metastases** | ***p*-value** |
| --- | --- | --- | --- |
| **Mean age years (SD)** | 67.05 (11.62) | 66.28 (11.27) | <0.001 |
| **Mean number of metastatic sites, *n* (%)*** | 0.95 (0.76) | 1.26 (0.90) | <0.001 |
| **Number of metastases, *n* (%)*** |  |  |  |
| 0 | 10,059 (28.4) | 1,503 (21.5) | <0.001 |
| 1 | 18,219 (51.5) | 2,812 (40.2) |  |
| 2 | 6,105 (17.2) | 2,017 (28.9) |  |
| 3 | 1,027 (2.9) | 656 (9.4) |  |
| *Sum of number of metastases in bone, brain and lung  **SD,** standard deviation | | | |

**Supplementary Table S2.** Presence of metastases according to sex

| **Liver metastases by sex *n* (%)** |  |  | ***p*-value** |
| --- | --- | --- | --- |
| Men | 18,708 (51.1) | 3,931 (521) | 0.098 |
| Women | 17,929 (48.9) | 3,613 (47.9) |  |
| **Brain metastases by sex *n* (%)** |  |  |  |
| Men | 16,386 (52.1) | 6,284 (49.1) | <0.001 |
| Women | 17,929 (48.9) | 6,527 (50.9) |  |
| **Bone metastases by sex *n* (%)** |  |  |  |
| Men | 12,894 (49.4) | 9,881 (53.9) | <0.001 |
| Women | 13,200 (50.6) | 8,448 (46.1) |  |
| **Lung metastases by sex *n* (%)** |  |  |  |
| Men | 15,494 (51.8) | 6,972 (50.0) | 0.001 |
| Women | 14,427 (48.2) | 6,963 (50.0) |  |
| **SD:** standard deviation | | | |
